# Plants Used for the Traditional Management of Cancer in the Eastern Cape Province of South Africa: A Review of Ethnobotanical Surveys, Ethnopharmacological Studies and Active Phytochemicals

**DOI:** 10.3390/molecules26154639

**Published:** 2021-07-30

**Authors:** Idowu Jonas Sagbo, Wilfred Otang-Mbeng

**Affiliations:** School of Biology and Environmental Sciences, University of Mpumalanga, Private Bag X11283, Mbombela 1200, South Africa; Wilfred.Mbeng@ump.ac.za

**Keywords:** anticancer drugs, medicinal plants, cancer, Eastern Cape, South Africa

## Abstract

Cancer occurrence is rapidly increasing all over the world, including in developing countries. The current trend in cancer management requires the use of herbal remedies since the majority of anticancer drugs are known to be costly, with unwanted side effects. In the Eastern Cape province, the use of medicinal plants for cancer management has been climbing steadily over the past two decades due to their cultural belief, low cost, efficacy, and safety claims. With the aim of identifying some potential anticancer plants for probable drug development, this study was undertaken to review plants reported by ethnobotanical surveys in the Eastern Cape province of South Africa for the traditional management of cancer. Information regarding plants used for cancer management in the Eastern Cape province was obtained from multidisciplinary databases and ethnobotanical books. About 24 plant species belonging to twenty families have been reported to be used for the traditional management of cancer in the Eastern Cape province. Among the anticancer plant species, only 16 species have been explored scientifically for their anticancer activities. This review authenticated the use of anticancer plant species in the Eastern Cape province and, therefore, identified several promising unexplored species for further scientific evaluation.

## 1. Introduction

Cancer, a generic term for a large group of diseases, may affect any part of the body. It is one of the world’s most horrifying diseases triggered by uncontrolled cellular proliferation. The development and progression of cancer are caused by an oncogene, the tumor suppressor gene (TSG), and alterations of the microRNA gene [1]. These genes act in an antagonistic manner to regulate a wide range of normal cellular activities, including cell survival metabolism and proliferation [1]. Mutations in these genes trigger the development of chronic and life-threatening diseases, particularly of cancer. Factors such as lifestyle, environment and nutrition have also been linked to the pathogenesis of cancer [2]. In a normal cell, the cell growth and division occur in a well-organized manner, but in cancer cells, defect-mediated cell death (apoptosis) leads to increased cell proliferation [3]. In addition, cancer cells grow as clumps, undergo uncontrolled cell division and display immortal traits. Currently, there are more than 100 types of cancer, including lung, prostate, colon, skin, breast cancers [4]. Interestingly, the types of cancer are typically named after the organ or tissue where they arise from or after the type of cells that form them. For example, cancer that begins in the cells of a lung and spreads to the liver is always referred to as lung cancer while brain cancer starts in the cells of the brain. The signs and symptoms of cancer vary according to the type or may not occur at all.

### 1.1. Cancer: A Global Concern

Cancer is increasingly a global health burden that has caused an intolerable number of deaths worldwide. It is one of the most horrific diseases of the twenty-first century, with approximately 6 million cases reported annually [5]. The International Agency for Research on Cancer (IARC) reported that there were 18.1 million new cancer cases and 9.5 million cancer-related deaths worldwide in 2018 [6]. The IARC also projected that the number of new cancer cases per year will reach 29.5 million and the number of cancer-related deaths is expected to reach 16.4 million by 2040 [6]. Globally, Australia tops the list of countries with the highest number of cancer rates, followed by New Zealand and Ireland [7]. Recently, studies have found that developing countries, in particular those in Africa, are facing an increasing incidence of cancer [8]. This is attributed to the aging and growth of the population as well as increased occurrence of risk factors linked with economic transition, including physical inactivity, obesity, reproductive behaviors and smoking [8]. In South Africa, there were about 108,168 new cancer cases and 56,802 deaths in 2020 among all ages [9]. Prostate cancer (25.8%) has recently been reported to be the number one cancer diagnosed among South African men, followed by lung cancer (12%), colon/rectum cancer (7.3%) and Kaposi sarcoma (4.9%), while in women, the most prevalent type is breast cancer (27.1%), followed by cervix uteri (18.7%), colorectum (6.3%), lung (4.9%) and corpus uteri (3.9%) cancers [9]. Although the burden of cancer in South Africa has increased, cancer continues to be a relatively low public health priority. This is mainly because of limited resources and other unrelenting public health challenges, including tuberculosis, diabetes, cerebrovascular diseases and communicable diseases.

### 1.2. Anticancer Drugs and Their Side Effects

In recent years, there have been several anticancer agents or drugs used for the management of cancer. These existing anticancer agents are divided into several categories, namely alkylating agents, antibiotics, antimetabolites, mitotic inhibitors, platinum compounds, biological response modifiers and hormone therapeutics. Table 1 shows the cancer types these anticancer agents are typically used for. A detailed description of the different types of these anticancer agents along with their potential side effects is provided below.

Alkylating agents are genotoxic drugs used for the treatment of cancer. They affect the nucleic acids and their function, thereby binding to the DNA, intervening with replication and transcription resulting in mutations [10]. The primary goal of alkylating agents is to trigger DNA damage in cancerous cells, causing them to enter apoptosis. These agents work by three distinctive mechanisms: (1) the attachment of alkyl groups to DNA bases, which in turn causes DNA to be fragmented by repair enzymes in a process to replace the alkylated bases, (2) alkylating agents causing the formation of cross-bridges, bonds between atoms in the DNA, thereby preventing DNA from being separated for synthesis or transcription and (3) they cause the mispairing of nucleotides, thereby leading to mutations [11]. However, due to the genetic damage caused by this class of drugs, the treatment of cancer patients with alkylating agents has been reported to be associated with an increased risk of secondary cancers [11]. Reports have also shown that alkylating agents are linked with gastrointestinal side effects and dose-limiting bone marrow toxicity [12]. Examples of alkylating agents include melphalan, bendamustine, cisplatin and dacarbazine.

Antibiotics used for the treatment of cancer are chemicals produced by microorganisms with anticancer activity [13]. These are mainly peptides and anthraquinones that inhibit uncontrolled proliferation, aggressive growth and metastatic activity of malignant cancers. They work by interacting with DNA in a variety of ways, including squeezing between the base pairs (intercalation), DNA strand breakage and inhibiting enzyme topoisomerase II [14]. They can kill cells throughout the proliferation cycle, even G0 phase cells, thereby fulfilling the antiproliferation capability of cancer cells by affecting the cell cycle [15]. However, a recent report indicated that antibiotics induce apoptosis of cancer cells, thereby targeting apoptotic gene B cell lymphoma-2 (Bcl-2), apoptotic pro-Bcl-2-associated x gene (Bax) and cancer suppressor gene P53, thus promoting cancer cell apoptosis in patients [13]. Antibiotic anticancer drugs are classified into different groups. These include anthracyclines, bleomycin, Adriamycin, dactinomycin and mithramycin. Anthracyclines are among the most important antibiotic anticancer drugs used to treat a variety of cancers. One of the main concerns when administering anthracyclines is that they can cause permanent damage to the heart if administered in high doses [16]. Examples of anthracycline antibiotic drugs include doxorubicin (Adriamycin), epirubicin and mitoxantrone.

Antimetabolites are also known as cytotoxic agents, have been developed for more than 60 years and are regarded as a pillar of cancer chemotherapy. They work by interfering with nucleic acid synthesis, thus acting as false metabolites, which are incorporated into DNA strands or block essential enzymes, thereby preventing DNA synthesis [17], and if new DNA cannot be made, cells are incapable of division. Antimetabolites are similar in structure to natural metabolites or enzymatic substrates, but they cannot be used productively by the body. They are confounded with the metabolites they resemble and are treated in the cell in a manner similar to normal compounds. However, like most cytotoxic anticancer agents, antimetabolites are toxic to normal cells, particularly to those in the gastrointestinal tract and bone marrow [12]. In addition, another report also indicated that antimetabolites cause side effects of immunosuppression, severe nausea and vomiting [18]. Some of the antimetabolite drugs from this class include gemcitabine, methotrexate and 5-fluorouracil (5-FU).

Mitotic inhibitors are another type of anticancer drugs used to treat several cancer types including lung cancer, breast cancer and lymphomas [19]. They inhibit mitosis (cell division) by disrupting microtubules, which are structures that separate the cell when it divides. They are frequently plant alkaloids and other compounds derived from natural products. Mitotic inhibitors work by discontinuing mitosis in the M phase of the cell cycle, but can damage cells in all phases, thus preventing enzymes from creating the proteins required for cell reproduction [20]. Some of the potential reported side effects of mitotic inhibitors include abdominal pain, weakness, back pain and lack of appetite [21]. Some of the examples of mitotic inhibitor drugs include vinblastine, paclitaxel and docetaxel.

Platinum compounds are another class of drugs used for cancer therapy. About half of the patients on chemotherapy are given platinum drugs [22]. The report also indicated that platinum drugs make up about 32 of 78 treatment regimens (combined with other drugs) [23]. They work by covalently binding to DNA, causing intra- and interstrand DNA adducts, thus resulting in the restriction of DNA replication, transcription, cell cycle arrest and programmed cell death [24]. The platinum drugs currently used with marketing approval in different parts of the world include carboplatin and cisplatin. While these drugs are effective, their use is limited by severe side effects [25,26]. For example, cisplatin is reported to be extremely nephrotoxic (damages the kidney) and also associated with severe nausea and vomiting in almost all patients [27]; carboplatin used in place of cisplatin is sometimes also reported to cause liver damage [26]. Oxaliplatin is reported to cause neurotoxic and pulmonary fibrosis (scar tissue in the lung connective tissue) [26].

Biological response modifiers (BRMs) have been reported to enhance the body’s ability to fight cancer through immune stimulation [28]. Several BRMs are generally accepted for the treatment of some types of cancer [29]. They can be both endogenous (produced naturally in the body) and exogenous (pharmaceutical drugs), and they can either improve or suppress the immune response. The BRMs work by blocking or reversing the processes that change normal cells or precancerous cells into cancer cells [30]. Furthermore, they increase the body’s capacity to repair or replace damaged or destroyed cells by other forms of cancer treatment, such as chemotherapy [30]. Similar to other forms of cancer treatments, BRMs also cause certain side effects depending on the type of treatment, though they slowly disappear once treatment is stopped. Typical side effects of BRMs include fever, fatigue, chills, nausea, back and joint pain [31]. Examples of BRMs are cytokines, interleukins, monoclonal antibodies (MAbs) and interferons.

Hormone therapy is another form of cancer treatment that is mainly used to treat specific types of breast cancer and prostate cancer that depend on sexual hormones to grow. Furthermore, several other cancers have also been reported to be treated with hormone therapy [32]. Hormone therapy works by stopping or slowing down the body from making hormones [32]. Since hormone therapy blocks the body’s capacity to produce hormones or interferes with how hormones behave, it can lead to undesirable side effects. Side effects always depend on the type of hormone therapy and the reaction of the body to it. Some side effects also vary between males and females. Some side effects reported in some men who use hormone therapy for the treatment of prostate cancer include nausea, fatigue, diarrhea, hot flashes and loss of interest in or ability to have sex [33] while the reported side effects in women who use hormone therapy for the treatment of breast cancer include loss of interest in sex, vaginal dryness, mood changes and nausea [34]. Examples of hormone therapy drugs include anastrozole, Zoladex, Trelstar and letrozole.

**Table 1:** Anticancer agents and the cancer types they are used to treat.

S/N	Anticancer Agents	Treated Cancer Type	References
1	Alkylating	Lung, ovarian, breast, multiple cancers and myeloma sarcoma	[21]
2	Antibiotics	Prostate, colorectal, ovarian, breast and lung cancers	[13]
3	Antimetabolites	Leukemias, breast, ovary, pancreatic and intestinal tract cancers	[21,35]
4	Mitotic inhibitor	Breast, lung, ovarian cancers, myeloma, lymphoma and leukemia	[36]
5	Platinum compound	Testicular, ovarian, lung, bladder, head and neck and colon cancers	[37]
6	Biological response modifier	Breast (trastuzumab), non-Hodgkin lymphoma and malignant melanoma	[28]
7	Hormone therapies	Breast, prostate and endometrial (uterine) cancers	[38,39]

### 1.3. Medicinal Plants for the Treatment of Cancer

**Figure 1:**
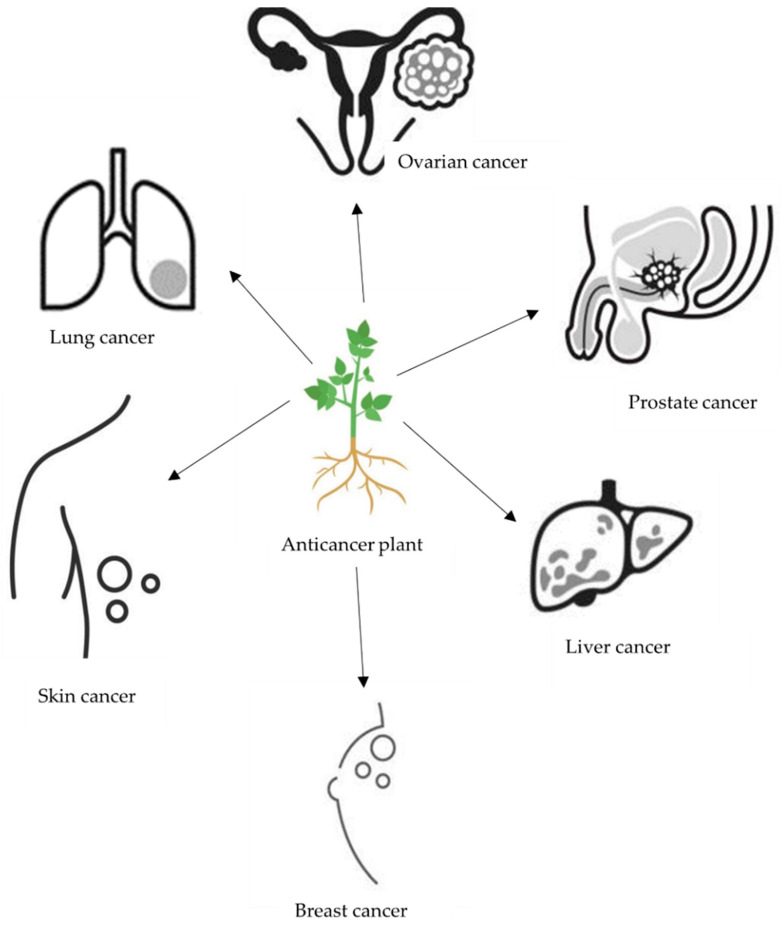
Illustration of anticancer plant use against some cancers.

In line with some limitations linked with the use of current synthetic anticancer agents, the use of medicinal plants for the treatment of cancer has been greatly accepted as part of medical interventions. This is mainly due to their fewer probable side effects, lesser costs and effectiveness, with several chemical compounds for the discovery of novel active substances against cancer [40,41]. Plants have always been an exemplary source of many of the currently available anticancer drugs. Almost two-thirds of the anticancer drugs are extracted from plants. For examples, vinblastine and vincristine (class: antimitotic drugs) used as anticancer drugs are derived from *Catharanthus roseus* (vinca plants) [42]. In general, plants continue to play a tremendous role in providing newer drugs and as such are a reservoir of natural chemicals which may provide chemoprotective ability against cancer. It is scientifically evident that several medicinal plants possess anticancer properties against a wide-ranging spectrum of cancers (Figure 1), thus overwhelming cancer-stimulating enzymes, inducing antioxidant effects, repairing DNA damage and increasing body immunity [43,44]. These anticancer properties of medicinal plants have been attributed to the active chemical constituents’ presence in these plants [45].

The search for medicinal plants for the treatment of cancer is still currently ongoing worldwide. Several review reports on plants with anticancer activity from different parts of the world have also been described in the literature [46,47,48]. In South Africa, many scientific researchers have reported several ethnobotanical surveys of plants used traditionally for the treatment of cancer [49,50]. A review by Chota et al. [51] reported the potential treatment of breast and lung cancer with *Dicoma anomala*. Another study conducted by Mfengwana [50] reported some traditional plants used for the treatment of cancer by South African communities. Despite the extensive use of these plants in the treatment of this deadly disease, there are still numerous indigenous medicinal plants that need urgent scientific documentation before they are permanently lost to future generations. It is in this context that this study focused on the Eastern Cape province, South Africa.

The Eastern Cape province is one of the poorest South African provinces, with the highest provincial unemployment rate (55%), and the majority of the population are rural dwellers, thereby they tend to rely heavily on medicinal plants for the treatment of diseases, including cancer. This is primarily because of their cultural beliefs, low cost, efficacy and safety claims of medicinal plants [52,53]. The Eastern Cape province is mostly inhabited by the isiXhosa-speaking people of Cape Nguni ancestry, and the use of medicinal plants for the treatment of diseases is an essential part of their cultural life, and this is not likely to change in the years to come. Thus, the current study was undertaken to review the plants reported in an ethnobotanical survey in the Eastern Cape province of South Africa for the traditional management of cancer and anticancer studies with active phytochemicals. This review study is anticipated to identify the current knowledge gap and serve as a vital baseline for future research. In addition, this study will further provide insight into scientifically underexploited plant species for future studies.

## 2. Results and Discussion

### 2.1. Plants Used in the Eastern Cape Province with the Anticancer Potential

**Figure 2:**
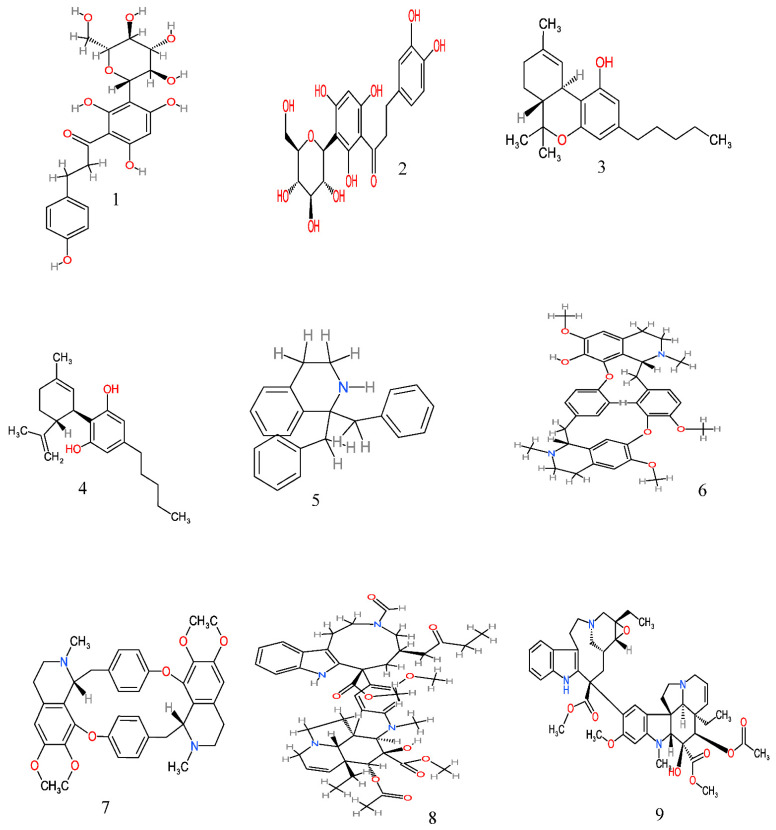

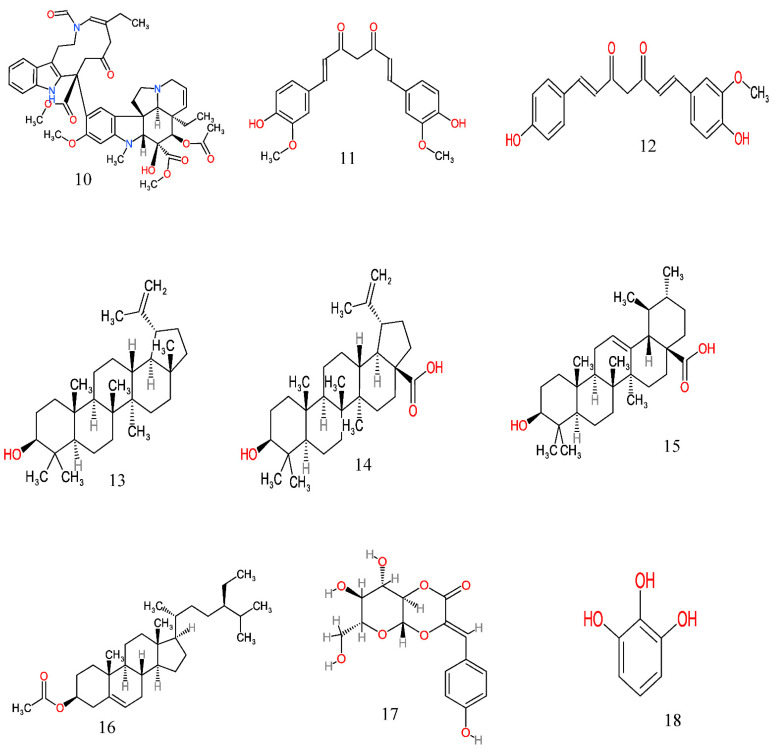

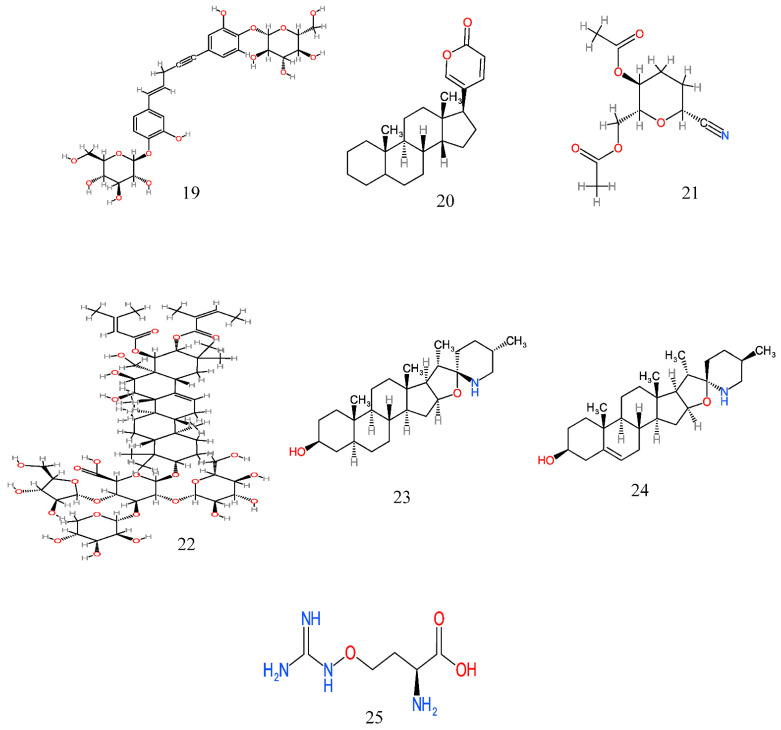
Anticancer molecules reported in some anticancer medicinal plants used in the Eastern Cape province, South Africa. The numbers 1–25 match the molecules reported in Table 3.

From the ethnobotanical survey, twenty-four plant species belonging to twenty botanical families have been claimed as anticancer plants by the people of Eastern Cape province (Table 2), though some of these plant species have been scientifically explored to justify their traditional usage. Only eight are yet to be scientifically investigated for their anticancer activity (Table 3). However, many of these anticancer plants have also been reported to possess several biological activities (Table 4). A comprehensive description of some plants used in Eastern Cape for the management of cancer with their anticancer activities, and their active phytochemicals (Figure 2) is as follows.

#### 2.1.1. *Aspalathus linearis*

*Aspalathus linearis* is an erect and highly variable shrub up to 2 m in height that belongs to the family Fabaceae [54]. The leaves of the plant are green and needle-like, about 15–60 mm long and 1 mm thick. *A. linearis* is easily dispersed in the winter rainfall area, predominantly in the Western Cape, Northern Cape and Eastern Cape provinces of South Africa [54]. In traditional medicine, the leaves are boiled and drunk as a tea for the treatment of cancer [55]. In addition, the plant is also used as an ingredient in cosmetics [54]. Literature surveys have shown that extracts from *A. linearis* have been reported to inhibit cell proliferation, thereby interfere with the growth of cancerous cells in the skin [56]. Furthermore, compounds (aspalathin and nothofagin) isolated from the methanol extract of *A. linearis* have also been reported to exhibit anticancer activity [57].

#### 2.1.2. *Agapanthus africanus*

*Agapanthus africanus* belongs to the family Agapanthaceae [58]. It is one of the most well-known garden plants in South Africa. *A. africanus* is an evergreen perennial plant that produces a leaf rosette of about 1 m in height from an underground bulb [58]. The leaves of the plant are green and strap-like, approximately 15-mm-wide, with an average length of 350 mm. *A. africanus* is found in the Eastern Cape and Western Cape provinces of South Africa [58]. Traditionally, powdered dried root of the plant is infused in water and then taken orally to treat cancer in the Eastern Cape province [59]. In addition, the roots of the plant are also used in the treatment of intestinal pain and heart troubles [60]. The literature survey showed no scientific studies on its anticancer properties.

#### 2.1.3. *Cannabis sativa*

*Cannabis sativa* is an erect annual plant up to 4 m tall with leaves alternate and palmately compound. The plant belongs to the family Cannabaceae and is widely distributed in Southern African countries, including South Africa and Botswana [61]. In South Africa, it is distributed in the Eastern Cape, Western Cape, Mpumalanga and KwaZulu–Natal provinces. In the Eastern Cape province, the crushed leaves of the plant are administered orally to treat cancer patients [61]. However, several scientific researchers have confirmed the anticancer activity of *C. sativa* and its compounds. Studies conducted by Bala et al. [62] revealed that the dichloromethane extract of *C. sativa* effectively inhibits growth and progression of breast cancer cells, with the IC_50_ value of 27.8 ± 5.0 μg/mL. Another study reported by Tariz and Reyaz [63] also showed that the acetone extract of the plant effectively exhibits inhibition against the glioblastoma (SF-268), the colon adenocarcinoma (HT-29) and breast adenocarcinoma (MCF-7) cells. The compound cannabidiol (CBD) isolated from *C. sativa* has also been reported to exhibit anticancer activity. CBD showed antiproliferative effects against breast cancer cells through various mechanisms, including apoptosis, autophagy and cell cycle arrest [64].

#### 2.1.4. *Catharanthus roseus*

*Catharanthus roseus* is a species of a flowering plant that belongs to the family Apocynaceae. It is an evergreen herbaceous or subshrub plant with a height of approximately 1 m. The leaves of the plant range from oval to oblong, 2.5–9-cm-long and 1–3.5-cm-wide [65]. The plant is native to Madagascar and was brought to South Africa as a garden ornamental plant, but has since escaped cultivation to become invasive in many parts of South African provinces, including Eastern Cape [65]. The alkaloid extracts of the aerial parts of the plant are used to treat various forms of cancer, such as uterine and breast cancer [66]. The infusion of the leaf is also used to treat diabetes [67]. Several researchers have reported the anticancer activity of *C. roseus*. Harshini et al. [68] revealed that the aqueous leaf extract of *C. roseus* exhibits significant inhibition against the growth of breast cancer cells (MCF-7 cells) at the concentrations investigated in the study. A study conducted by Pham et al. [69] also found that the *C. roseus* root and stem extracts possess significant cytotoxic activity towards some cancer cell lines. The compound catharoseumine isolated from *C. roseus* was found to exhibit an inhibitory effect against the human promyelocytic leukemia HL-60 cell line, with the IC_50_ value of 6.28 μM [70]. In another study, some other compounds (vinamidine, leurosine and catharine) isolated from *C. roseus* have also been reported to possess an inhibitory activity against a human breast cancer cell line (MDA-MB-231), with the IC_50_ value range of 0.73–10.67 μM [71].

#### 2.1.5. *Eucomis autumnalis*

*Eucomis autumnalis* is a garden plant that belongs to the family Hyacinthaceae. It is a deciduous bulb that grows in summer. The bulbs of the plant are large, ovoid in shape, and give rise to a rosette of large broad leaves about 12–35 cm long and 7.5 cm wide [72]. The plant grows in open grassland and forest margins in the Eastern Cape and Limpopo provinces of South Africa [72]. In traditional medicine, the decoctions prepared from warmed bulbs in water or milk are usually administered orally to treat cancer by the people of the Eastern Cape province [59]. The decoctions are also used for the treatment of other ailments such as stomach ache, fevers, syphilis and urinary diseases [72]. The in vitro anticancer study reported by Bisi-Johnson et al. [73] revealed that the methanol extract of *E. autumnalis* exhibited a significant cytotoxic effect against a human hepatoma cell line (Huh-7), with the IC_50_ value of 7.8 μg/mL, as compared to berberine (IC_50_, 9.8 μg/mL), the positive control used in the study.

#### 2.1.6. *Euphorbia ingens*

*Euphorbia ingens* (family Euphorbiaceae) is an erect succulent tree that grows up to 12 m in height. The plant prefers hot areas and is able to survive in areas that experience long periods of drought [74]. It grows on rocky outcrops and is distributed across South African provinces, such as the North–West, Eastern Cape, KwaZulu–Natal and Limpopo provinces [74]. The Xhosa people in the Eastern Cape province traditionally used the latex of the plant and then applied it topically on external cancers every day [59]. In addition, the plant is also used medicinally as a purgative or for the treatment of ulcers [74]. Despite the use of *E. ingens* for the treatment of cancer, there is still a dearth of scientific information on its anticancer properties.

#### 2.1.7. *Hypoxis argentea*

*Hypoxis argentea* is one of the numerous species of the genus *Hypoxis*, the largest genus of the family Hypoxidaceae [75]. *H. argentea* is a perennial plant that is mostly found in grassland and on rocky outcrops. It has thin ribbed leaves with silky yellowish hairs and small yellow flowers [76]. The plant has mostly occurred in the Cape provinces of South Africa [76]. The fresh corms of the plant are stamped, boiled in water and then administered orally to treat cancer in the Eastern Cape [59]. The plant is also used in traditional veterinary practice for treating cracked cow teats and injuries in horses [76]. The literature survey revealed no report with regard to its anticancer activities.

#### 2.1.8. *Pittosporum viridiflorum*

*Pittosporum viridiflorum* is an evergreen tree that belongs to the family Pittosporaceae. The leaves of the plant are frequently wider above the middle and dark-green [77]. The plant grows in high forest and scrubs on forest margins and on-stream banks. It is widely dispersed in the Eastern part of South Africa [77]. The decoction of *P. viridiflorum* is prepared from boiled fruit, filtered and then administered orally for the treatment of cancer [59]. In addition, decoctions or infusions are also widely used in South Africa for abdominal pain and fever [77]. Several researchers have reported the anticancer properties of *P. viridiflorum* [78]. Madikizela and McGaw [79] reported the anticancer potential of acetone extracts of *P. viridiflorum* against human cancer cells (breast MCF-7, colorectal Caco-2, lung A549 and cervical Hela cells) tested, with the IC_50_ value ranging from 3.16 to 26.87 μg/mL. In the same study, the author also revealed that the ethanol extract of the plant exhibits a significant anticancer activity against cervix, breast and colorectal cancer cells, with the IC_50_ values ranging from 13.28 to 23.37 μg/mL. Another study reported by Poschner [80] revealed that the methanol extract of *P. viridiflorum* exhibited a cytotoxic effect against HL-60 leukemia cells, with the IC_50_ value of 5.15 μg/mL.

#### 2.1.9. *Solanum aculeastrum*

*Solanum aculeastrum* is a small tree approximately 1–5-m-high, with lobed discolorous leaves. The plant belongs to the family Solanaceae. *S. aculeastrum* occurs naturally in grassland, woodland and on forest margins, but is dispersed in Eastern Cape, Mpumalanga, Limpopo, KwaZulu–Natal and Western Cape [81]. The decoction prepared from boiled fruit is filtered and then administered orally for the treatment of breast cancer in Eastern Cape [59]. The methanol and aqueous fruit extracts of *S. aculeastrum* have been reported to exhibit antiproliferative activity against three human tumor cell lines (HeLa, MCF7 and HT29), with the IC_50_ value ranging between 17.1 and 48.3 μg/mL [82]. Another study conducted by Burger et al. [83] also revealed that both the crude extract and the aqueous fraction of *S. aculeastrum* exhibit a significant cytotoxic effect against some cancerous cells, such as A2780 ovarian carcinoma, DU145 prostate carcinoma and Sk-Br3 breast adenocarcinoma. Koduro et al. [84] also reported that tomatidine and solasodine isolated from the berries of *S. aculeastrum* inhibit the growth of cancer cell lines (HeLa, MCF7and HT29) by blocking the cell cycle in the G0/G1phase after 24-h exposure to the compounds.

#### 2.1.10. *Sutherlandia frutescens*

*Sutherlandia frutescens* is an attractive shrub of up to about 1 m in height that belongs to the family Fabaceae. The leaves of the plant are pinnately compound and grey–green in color. The plant naturally occurs in Southern African countries, such as South Africa, Namibia and Botswana [67]. In South Africa, the plant is widely found in the Eastern Cape, Western Cape, KwaZulu–Natal and Mpumalanga provinces [67]. In traditional medicine, the decoctions of the plant prepared from all the plant parts are administered orally to treat cancer [67]. In addition, the plant is now increasingly used as an immune enhancer for the treatment of HIV/AIDS [85]. The literature survey revealed that several researchers have investigated the anticancer activity of *S. frutescens*. Research conducted by Gouws et al. [86] showed that the aqueous extract of *S. frutescens* decreased LS180 colorectal cell growth and viability, with the IC_50_ value of 2.63 mg/mL, as compared to paclitaxel, the positive control used in the study. Another study by Chinkwo [87] also revealed that the crude aqueous extract of *S. frutescens* showed significant cytotoxicity against neoplastic (cervical carcinoma) cells. A separate study conducted by Motadi [88] also reported that the *S. frutescens* methanol extract induced growth inhibition of human squamous carcinoma (SiHa cell line), with the IC_50_ value of 50 μg/mL. In the same study, the author also indicated that the extract induced cell cycle arrest at the S phase.

**Table 2:** Plants used for the treatment of cancer in the Eastern Cape province.

S/N	Scientific Name	Local Name	Family	Part Used	Conservation Status	Mode of Preparation (Administration)	Cancer Type Suppressed	References
1	*Aloe ferox* Mill	IKhala	Xanthorrhoeaceae	Leaf sap, leaves and roots	NT, NE	The sap is applied topically to treat skin cancer	Skin cancer	[89]
2	*Aspalathus linearis*	Inkanga	Fabaceae	Leaves	NT, E	The leaves are boiled and drunk as tea	Cervical cancer	[55]
3	*Agapanthus africanus* (L.) Hoffmanns	Mathunga	Agapanthaceae	Root	NT	Dried roots are powdered and infused in water and then taken orally	Uterine and breast cancers	[90]
4	*Cannabis sativa* L.	Umya	Cannabaceae	Leaves	NT, NE	Crushed leaves of the plant are administered orally	Skin cancer	[91]
5	*Catharanthus roseus* (L.)	*	Apocynaceae	Leaves, whole plant	NT, NE	The extract from the aerial part is administered orally	Breast, lung and uterine cancers	[92]
6	*Celtis africana* Burm.f.	UmVumvu	Cannabaceae	Bark and leaves	NT, NE	Dried bark and roots of the plant are pulverized and infused in milk and then taken orally	Unspecified	[59]
7	*Cissampelos* capensis L.	Umayisake	Menispermaceae	Root	NT, NE	The root is used as a paste and applied directly	Skin and stomach cancers	[93]
8	*Curcuma longa*	Tumeric	Zingiberaceae	Root	NT, NE	The infusion of the root is taken orally	Unspecified	[55]
9	*Curtisia dentata* (Burm.f.) C.A.Sm.	UmLahleni, UmGxina	Cornaceae	Bark and leaves	NT, NE	A decoction is prepared from boiled bark and roots and administered orally	Esophageal cancer	[94]
10	*Elytropappus rhinecerotis*	*	Asteraceae	Whole plants	NT, E	Infusions of young branches in brandy or wine	Stomach cancer	[95]
11	*Eucomis autumnalis* (Mill.) Chitt	Umathunga	Hyacinthaceae	Bulbs	V, NT	Decoctions are prepared from warmed bulbs and taken orally	Unspecified	[59]
12	*Euphorbia ingens* E.Mey. ex Boiss	Nkondze	Euphorbiaceae	Latex	NT, NE	Latex is applied topically on external cancers every day	Skin cancer	[59]
13	*Gunnera perpensa* L.	Ighobo	Gunneraceae	Rhizomes	NE	A decoction or infusion is prepared from the rhizome and taken orally	Unspecified	[92]
14	*Hermannia depressa*	Seletjana	Malvaceae	Leaves, roots	NT, NE	Crushed leaves are used to treat cancer	Unspecified	[96]
15	*Hypoxis argentea* Harv. ex Baker	Inongwe	Hypoxidaceae	Corms	NT, NE	Fresh corms are boiled in water and then administered orally	Unspecified	[59]
16	*Hypoxis hemerocallidea* Fisch., C.A.Mey. and Avé-Lall	Ilabatheka, Ilabatheka	Hypoxidaceae	Corms	NT, NE	Pulverized corms are boiled in water and taken orally	Prostate cancer	[59,75]
17	*Knowltonia capensis* (L.) Huth	*	Ranunculaceae	Leaves	V, E	Crushed leaves are prepared as poultices and applied directly on external tumors	Skin cancer	[59]
18	*Merwilla plumbea* (Lindl.) Speta	Umasixabane, Ugontsana	Hyacinthaceae	Bulbs	NT, NE	Decoctions are prepared from warmed bulbs and taken orally	Unspecified	[59]
19	*Melianthus major* L.	Ubutyayi	Melianthaceae	Leaves	NT, E	Decoctions are prepared from leaves and then administered orally	Unspecified	[97]
20	*Pittosporum viridiflorum* Sims	Umgqwengqwe	Pittosporaceae	Bark and root	NT, NE	Infusions are prepared from stamped bark and roots and then administered orally	Unspecified	[59]
21	*Sarcophyte* sanguinea Sparrm. subsp. *sanguinea*	*	Balanophoraceae	Whole plants	NT, NE	A decoction from the whole plant is administered orally	Unspecified	[97]
22	*Solanum aculeastrum* Dunal subsp. *aculeastrum*	Itunga, Umthuma	Solanaceae	Fruits and leaves	NT, NE	A decoction is prepared from boiled fruit is filtered and then administered orally	Breast cancer	[98]
23	*Sutherlandia frutescens* L. R.Br.	Umnwele	Fabaceae	leaves, flower and seed	V, NE	Decoctions are prepared from all the plant parts and administered orally	Colorectal cancer	[59,86]
24	*Tulbaghia violacea* Harv	Utswelane	Alliaceae	Leave, bulb	NT, E	The fresh bulbs are boiled in water and the decoctions are taken orally	Esophageal cancer	[92,99]

NT: neither rare nor threatened; V: vulnerable; E: endemic; NE: nonendemic, *: not available.

**Table 3:** Reported anticancer activity of medicinal plants used in the Eastern Cape province, South Africa.

S/N	Scientific Name	Plant Part Used	Extract	Active Phytochemical	Effect on Cancer Cells/Anticancer Activity	References
1	*Aloe ferox* Mill	Leaves	Dichloromethane	*	Inhibits a prostate cancer (PC3) cell line	[100]
2	*Aspalathus linearis*	Whole plant	Aqueous, methanol	Nothofagin (**1**) and aspalathin (**2**)	Inhibits cell proliferation; thus, interferes with the growth of cancerous cells in the skin	[56]
3	*Agapanthus africanus* (L.) Hoffmanns	*	*	*	*	*
4	*Cannabis sativa* L.	Stem, fruit and leaf	Dichloromethane, methanol and acetone	Delta-9-tetrahydrocannabinol (THC) (**3**) and cannabidiol (CBD) (**4**)	Inhibits the growth of breast adenocarcinoma (MCF-7), glioblastoma (SF-268) and colon adenocarcinoma (HT-29) cells	[62,63]
5	*Celtis africana* Burm.f.	*	*	*	*	*
6	*Cissampelos capensis* L.	Rhizomes	Alkaloid	Bisbenzyltetrahydroisoquinoline (**5**), 12-O-methylcurine (**6**), and cycleanine (**7**)	Inhibits the growth of breast adenocarcinoma (MCF7), melanoma (UACC62) and renal (TK10) cells	[93,101]
7	*Catharanthus roseus* (L.)	Leaves, stem	Alkaloid, aqueous	Vinamidine (**8**), leurosine (**9**) and catharine (**10**)	Cytotoxicity against ovarian (A2780), lung (H460), skin (A431), prostrate (Du145), colon (HT29 and) breast (MCF-7 and MDA-MB-231) cell lines	[69]
8	*Curcuma longa*	Leaves, rhizomes	Aqueous	Curcumin (**11**) and desmethoxycurcumin (**12**)	Inhibits the proliferation/viability of human lung cancer (A549), colon cancer (HT29), glioblastoma (T98G) and Chinese hamster ovary (CHO) cell lines	[102,103]
9	*Curtisia dentata* (Burm.f.) C.A.Sm.	Leaves	Acetone	Lupeol (**13**), betulinic acid (**14**), ursolic acid (**15**) and β-sitosterol (**16**)	Inhibits the growth of human breast adenocarcinoma (MCF7), human cervical cancer cells (Hela), human colorectal carcinoma cells (caco-2) and human hepatocellular carcinoma cells (HepG2)	[104,105]
10	*Elytropappus rhinecerotis*	*	*	*	*	*
11	*Eucomis autumnalis* (Mill.) Chitt	Root	Methanol	*	Inhibits the growth of a human hepatoma cell line (Huh-7)	[73]
12	*Euphorbia ingens* E.Mey. ex Boiss	*	*	*	*	*
13	*Gunnera perpensa* L.	Root	Dichloromethane	Z-venusol 5 (**17**) and pyrogallol (**18**)	Cytotoxicity against prostate (PC3) and breast cancer (MCF-7) cell lines	[50]
14	*Hermannia depressa*	Shoot	Acetone and aqueous	*	Inhibition of breast cancer (MCF-7) and cervical cancer HeLa cell lines	[96]
15	*Hypoxis argentea* Harv. ex Baker	*	*	*	*	*
16	*Hypoxis hemerocallidea* Fisch., C.A.Mey. and Avé-Lall	Corms	Chloroform	Hypoxoside (**19**)	Inhibits the growth of colon adenocarcinoma (HT-29), human cervical cancer cells (Hela) and breast adenocarcinoma (MCF7) cells causing DNA cell cycle arrest at the late G1 and/or early S phase	[106]
17	*Knowltonia capensis* (L.) Huth	*	*	*	*	*
18	*Merwilla plumbea* (Lindl.) Speta	*	*	*	*	*
19	*Melianthus major* L	Leaves	Petroleum ether, chloroform, ethyl acetate and methanol	Bufadienolide (**20**) and 2β-acetoxy-3,5-di-O-acetylhellebrigenin (**21**)	Cytotoxicity towards human epithelial larynx carcinoma (Hep 2) and breast cancer (MCF-7) cell lines	[107,108]
20	*Pittosporum viridiflorum* Sims	Leaves and bark	Acetone, methanol	Pittoviridoside (**22**)	Inhibits the growth of breast MCF-7, colorectal Caco-2, lung A549, cervical Hela and ovarian cancer A2780 cells	[78,80]
21	*Sarcophyte* sanguinea Sparrm. subsp. *sanguinea*	*	*	*	*	*
22	*Solanum aculeastrum* Dunal subsp. *aculeastrum*	Fruit	Methanol and aqueous	Tomatidine (**23**) and solasodine (**24**)	Antiproliferative activity against cervical HeLa, breast adenocarcinoma (MCF7) and colon adenocarcinoma (HT29) cells by blocking the cell cycle in the G0/G1phase; cytotoxic effect against A2780 ovarian carcinoma, DU145 prostate carcinoma and Sk-Br3 breast adenocarcinoma cells	[83,84]
23	*Sutherlandia frutescens* L. R.Br.	Whole plant	Methanol and aqueous	L-canavanine (**25**)	Induces apoptosis and cytotoxicity in neoplastic cells (cervical carcinoma) and CHO (Chinese hamster ovary cells) cell lines; induces growth inhibition of human squamous carcinoma (SiHa cell line) thereby causing cell cycle arrest at the S phase	[87,88]
24	*Tulbaghia violacea* Harv	Leaves	Methanol, hexane, acetone, butanol	*	Induces apoptosis in breast cancer (MCF7 and MB MDA231), cervical cancer (HeLa and ME-180) and oral cancer (KB) cell lines causing cell cycle arrest at the G2/M phase	[88]

*: not available.

**Table 4:** Pharmacological activities of anticancer plant species used in Eastern Cape.

S/N	Scientific Name	Other Reported Pharmacological Activities	References
1	*Aloe ferox* Mill	Anti-inflammatory, antioxidant and antimicrobial	[109,110,111]
2	*Aspalathus linearis*	Antiviral, antioxidant and anti-inflammatory	[112,113]
3	*Agapanthus africanus* (L.) Hoffmanns	Antifungal	[114]
4	*Cannabis sativa* L.	Antimicrobial and antioxidant	[115]
5	*Catharanthus roseus* (L.)	Antimicrobial, antioxidant, anthelmintic, antifeedant, anti-sterility, antidiarrheal, antidiabetic	[116,117,118]
6	*Celtis africana* Burm.f.	Antioxidant and anti-inflammatory	[119,120]
7	*Cissampelos capensis* L.	Antimicrobial	[121]
8	*Curcuma longa*	Antioxidant, anti-inflammatory, antimicrobial, hepatoprotective effect, exhibits protective effects against gastrointestinal tract cancer	[122,123,124]
9	*Curtisia dentata* (Burm.f.) C.A.Sm.	Antimicrobial and antioxidant	[125]
10	*Elytropappus rhinecerotis*	Antimicrobial	[126]
11	*Eucomis autumnalis* (Mill.) Chitt	Antibacterial and antioxidant	[73]
12	*Euphorbia ingens* E.Mey. ex Boiss	Antimicrobial	[127]
13	*Gunnera perpensa* L.	Antibacterial, antifungal, anti-inflammatory, antinociceptive and antioxidant	[128,129,130]
14	*Hermannia depressa*	Anti-inflammatory and antibacterial	[131]
15	*Hypoxis argentea* Harv. ex Baker	Antioxidant and antidiabetic	[132,133]
16	*Hypoxis hemerocallidea* Fisch., C.A.Mey. and Avé-Lall	Anticonvulsant, antiuropathogenic, antioxidant, antidiabetic and antibacterial	[134,135]
17	*Knowltonia capensis* (L.) Huth	Antibacterial	[136]
18	*Merwilla plumbea* (Lindl.) Speta	Antimicrobial, antioxidant, antifungal	[137,138]
19	*Melianthus major* L	Antimicrobial and antioxidant	[108,139]
20	*Pittosporum viridiflorum* Sims	Antidiarrheal, antimalarial, anti-inflammatory, antioxidant and antimicrobial	[78]
21	*Sarcophyte* sanguinea Sparrm. subsp. *sanguinea*	Antimicrobial	[140]
22	*Solanum aculeastrum* Dunal subsp. *aculeastrum*	Antimicrobial and antioxidant	[82,98]
23	*Sutherlandia frutescens* L. R.Br.	Antibacterial, antioxidant, anti-inflammatory and antidiabetic	[141,142,143]
24	*Tulbaghia violacea* Harv	Antibacterial, antifungal, antioxidant and antidiabetic	[144,145]

## 3. Materials and Methods

A comprehensive literature search was thoroughly conducted from December 2020 to May 2021. Information about the plants used for the traditional management of cancer in the Eastern Cape province of South Africa was retrieved from various online databases, including Web of Science, Medline, Google Scholar, Science Direct, Scopus, PubMed, Medline, Web of Science and Library Search. Additionally, dissertations, theses, and ethnobotanical books were also retrieved from the libraries of universities. The keywords and terms used to search for the relevant articles, included “Eastern Cape”, “traditional medicine”, “cancers”, “ethnopharmacology” and “medicinal plants”. The scientific and common names of the plants were validated in reference to the PlantZAfrica [146] and the Plant List [147]. 

## 4. Conclusions and Recommendations

Cancer is a genetic condition in which certain cells of the body develop uncontrollably and spread to other parts of the body. Its incidence is rapidly increasing all over the world, including in developing countries such as South Africa. The current trend in cancer management requires the use of medicinal plants since the majority of anticancer drugs are known to be costly, with unwanted side effects. In the Eastern Cape province, the number of people using medicinal plants for the management of cancer has been rising steadily over the last two decades. This is attributed to their cultural beliefs, low cost, efficacy and safety claims of these medicinal plants. In this review study, out of the twenty-four medicinal plants reportedly used in the Eastern Cape province for the management of cancer, only sixteen plants have been scientifically studied for their anticancer activity, and many of these plants exhibited their anticancer activity through inhibition of the growth of several cancer cell lines or attenuate their proliferation. It is highlighted that the anticancer activity of these plants is mainly due to their different phytochemical compounds (Table 1). Hence, an effort needs to be devoted to the isolation and purification of these anticancer bioactive components. In addition, studies are also needed to highlight the mechanism of anticancer action of many previously explored and many unexplored plants.

## References

[1] Wanga L., Wu C., Rajasekaran N., Shin Y.K. (2018). Loss of tumor suppressor gene function in human cancer: An overview. Cell Physiol. Biochem..

[2] Anand P., Kunnumakara A.B., Sundaram C., Harikumar K.B., Tharakan S.T., Lai O.S., Sung B., Aggarwal B.B. (2008). Cancer is a preventable disease that requires major lifestyle changes. Pharm. Res..

[3] Sagbo I.J., Otang-mbeng W. (2020). Anti-proliferative and genotoxic activities of the *Helichrysum petiolare* Hilliard & B.L. Burtt. Sci. Pharm..

[4] (2021). National Cancer Institute. https://www.cancer.gov/about-cancer/understanding/what-is-cancer.

[5] Ma X., Yu H. (2006). Global burden of cancer. Yale J. Biol. Med..

[6] International Agency for Research on Cancer (IARC) Statistics at a Glance: The Burden of Cancer Worldwide. https://www.cancer.gov/about-cancer/understanding/statistics.

[7] World Cancer Research Fund International (WCRFI) Which Countries Have the Highest and Lowest Cancer Rates?. https://www.wcrf.org/dietandcancer/global-cancer-data-by-country/.

[8] Bahnassy A.A., Mona S., Abdellateif M.S., Zekri A.R.-N. (2020). Cancer in Africa: Is it a genetic or environmental health problem?. Front. Oncol..

[9] Globocon (2020). South Africa—Global Cancer Observatory. https://gco.iarc.fr/today/data/factsheets/populations/710-south-africa-fact-sheets.pdf.

[10] Guimarães I.D., Daltoé R.D., Herlinger A.L., Madeira K.P., Ladislau T., Valadão L.C., Junior P.C.M.L., Teixeira S.F., Amorim G.M., dos Santos D.Z. (2013). Conventional cancer treatment. Intech Open.

[11] Weber G.F. (2015). DNA damaging drugs. Molecular Therapies of Cancer.

[12] Scholar E. (2007). Alkylating agents. xPharm: The Comprehensive Pharmacology Reference.

[13] Gao Y., Shang Q., Li W., Guo W., Stojadinovic A., Mannion C., Man Y., Chen T. (2020). Antibiotics for cancer treatment: A double-edged sword. J. Cancer.

[14] Anticancer Drugs II. http://chemistry.elmhurst.edu/vchembook/655cancer2.html.

[15] Xiao Z., Sperl B., Ullrich A. (2014). Metformin and salinomycin as the best combination for the eradication of NSCLC monolayer cells and their alveospheres (cancer stem cells) irrespective of EGFR, KRAS, EML4/ALK and LKB1 status. Oncotarget.

[16] How Chemotherapy Drugs Work. https://www.cancer.org/treatment/treatments-and-side-effects/treatment-types/chemotherapy/how-chemotherapy-drugs-work.html.

[17] Walsh D. (2009). Palliative Medicine.

[18] Chortkoff B., Stenehjem D., Hemmings H.C., Egan T.D. (2019). Chemotherapy, immunosuppression, and anesthesia. Pharmacology and Physiology for Anesthesia: Foundations and Clinical Application.

[19] Saville M.W., Lietzau J., Pluda J.M., Wilson W.H., Humphrey R.W., Feigel E., Steinberg S.M., Broder S., Yarchoan R., Odom J. (1995). Treatment of HIV-associated Kaposi’s sarcoma with paclitaxel. Lancet.

[20] Jiang N., Wang X., Yang Y., Dai W. (2006). Advances in mitotic inhibitors for cancer treatment. Mini Rev. Med. Chem..

[21] Chemotherapy Types and the Cancers They’re Used for. https://www.healthline.com/health/cancer/chemotherapy-types.

[22] Galansk I.M., Jakupec M.A., Keppler B.K. (2015). Update of the preclinical situation of anticancer platinum complexes: Novel design strategies and innovative analytical approaches. Curr. Med. Chem..

[23] Ali A., McCrudden C., McCarthy H. (2017). Evaluation of the impact of nitric oxide on resistance to platinum-based chemotherapeutics. Nitric Oxide (Donor/Induced) in Chemosensitizing.

[24] Grant C.H., Gourley C. (2015). Relevant cancer diagnoses, commonly used chemotherapy agents and their biochemical mechanisms of action. Cancer Treatment and the Ovary.

[25] Waszkiewicz K. (2001). About side effects of platinum drugs. Postep. Hig. Med. Dosw..

[26] Oun R., Moussa Y.E., Wheate N.J. (2018). The side effects of platinum-based chemotherapy drugs: A review for chemists. Dalt. Trans..

[27] Pabla N., Dong Z. (2008). Cisplatin nephrotoxicity: Mechanisms andrenoprotective strategies. Kidney Int..

[28] Gupta S., Kanodia A.K. (2002). Biological response modifiers in cancer therapy. Natl. Med. J. India.

[29] Kuroki M., Miyamoto M., Morisaki T., Yotsumoto F., Shirasu N., Taniguchi Y., Soma G. (2012). Biological response modifiers used in cancer biotherapy. Anticancer Res..

[30] Darling R.G., Noste E.E. (2016). Future biological and chemical weapons. Ciottone’s Disaster Medicine.

[31] Hall S.J., Klotz L., Pantuck A.J., George D.J., Whitmore J.B., Frohlich M.W., Sims R.B. (2011). Integrated safety data from 4 randomized, double-blind, controlled trials of autologous cellular immunotherapy with sipuleucel-T in patients with prostate cancer. J. Urol..

[32] Hormone Therapy. https://www.cancer.org/treatment/treatments-and-side-effects/treatment-types/hormone-therapy.html.

[33] Hormone Therapy for Prostate Cancer. https://www.cancer.org/cancer/prostate-cancer/treating/hormone-therapy.html.

[34] Cella D., Fallowfield I.J. (2008). Recognition and management of treatment-related side effects for breast cancer patients receiving adjuvant endocrine therapy. Breast Cancer Res. Treat..

[35] Valenzuela M.M.A., Neidigh J.W., Wall N.R. (2014). Antimetabolite treatment for pancreatic Cancer. Chemotherapy.

[36] Rowinsky E.K., Donehower R.C. (1995). Pacilitaxel (taxol). N. Engl. J. Med..

[37] Köberle B., Schoch S. (2021). Platinum complexes in colorectal cancer and other solid tumors. Cancers.

[38] Carlson M.J., Thiel K.W., Leslie K.K. (2014). Past, present, and future of hormonal therapy in recurrent endometrial cancer. Int. J. Womens Heal..

[39] Fairchild A., Tirumani S.H., Rosenthal M.H., Howard S.A., Krajewski K.M., Nishino M., Shinagare A.B., Jagannathan J.P., Ramaiya N.H. (2015). “Hormonal therapy in oncology: A primer for the radiologist. Am. J. Roentgenol..

[40] Greenwell M., Rahman P.K.S.M. (2015). Medicinal plants: Their use in anticancer treatment. Int. J. Pharm. Sci. Res..

[41] IqbaI J., Abbasi B.A., Mahmood T., Kanwal S., Ali B., Shah S.A., Khalil A.T. (2017). Plant-derived anticancer agents: A green anticancer approach. Asian Pac. J. Trop. Biomed..

[42] Moudi M., Go R., Yien C.Y.S., Nazre M. (2013). Vinca alkaloids. Int. J. Prev. Med..

[43] Khan T., Ali M., Khan A., Nisar P., Jan S.H., Afrid S., Shinwari Z.K. (2020). Anticancer plants: A review of the active phytochemicals, applications in animal models, and regulatory aspects. Biomolecules.

[44] Padmaharish V., Lakshmi T. (2017). Anticancer activities of medicinal plants—An update. J. Pharm. Sci. Res..

[45] Choudhari A.S., Mandave P.C., Deshpande M., Ranjekar P., Prakash O. (2019). Phytochemicals in cancer treatment: From preclinical studies to clinical practice. Front. Pharmacol..

[46] Tesfaye S., Asres K., Lulekal E., Alebachew Y., Tewelde E., Kumarihamy M., Muhammad I. (2020). Ethiopian medicinal plants traditionally used for the treatment of cancer, Part 2: A review on cytotoxic, antiproliferative, and antitumor phytochemicals, and future Perspective. Molecules.

[47] Tyagi N., Sharma G.N., Shrivastava B., Saxena P., Kumar N. (2017). Medicinal plants: Used in anti-cancer treatment. Int. J. Res. Dev. Pharm. Life Sci..

[48] Kuruppu A.I., Paranagama P., Goonasekara C.L. (2019). Medicinal plants commonly used against cancer in traditional medicine formulae in Sri Lanka. Saudi Pharm. J..

[49] Twilley D., Rademan S., Lall N. (2020). A review on traditionally used South African medicinal plants, their secondary metabolites and their potential development into anticancer agents. J. Ethnopharmacol..

[50] Mfengwana P.H. (2019). Evaluation of Pharmacological Properties of Traditional Medicinal Plants Used for the Treatment of Cancer by South African and Lesotho Communities. Ph.D. Thesis.

[51] Chota A., George B.P., Abrahamse H. (2020). Potential treatment of breast and lung Cancer using *Dicoma anomala*, an African medicinal plant. Molecules.

[52] Erasto P., Adebola P., Grierson D., Afolayan A.J. (2005). An ethnobotanical study of plants used for the treatment of diabetes in the Eastern Cape province, South Africa. Afr. J. Biotechnol..

[53] Wilkinson K. (2018). FACTSHEET: South Africa’s Official Poverty Numbers. Africa Check. https://africacheck.org/factsheets/factsheet-south-africas-official-poverty-numbers/.

[54] Govender M. (2007). National Herbarium. http://pza.sanbi.org/aspalathus-linearis.

[55] Sewani-Rusike C.R., Mammen M. (2014). Medicinal plants used as home remedies: A family survey by first year medical students. Afr. J. Tradit. Complement. Altern. Med..

[56] Magcwebeba T.U., Swart P., Swanevelder S., Joubert E., Gelderblom W.C.A. (2016). In vitro chemopreventive properties of green tea, Rooibos and honeybush extracts in skin Cells. Molecules.

[57] Fantoukh O.I., Dale O.R., Parveen A., Hawwal M.F., Ali Z., Manda V.K., Khan S.I., Chittiboyina A.G., Viljoen A., Khan I.A. (2019). Safety assessment of phytochemicals derived from the globalized South African rooibos tea (*Aspalathus linearis*) through interaction with CYP, PXR, and P-gp. J. Agric. Food Chem..

[58] Jamieson R. (2004). Centre for Home Gardening, Kirstenbosch. http://pza.sanbi.org/agapanthus-africanus.

[59] Koduru S., Grierson D.S., Afolayan A.J. (2007). Ethnobotanical information of medicinal plants used for treatment of cancer in the Eastern Cape Province, South Africa. Curr. Sci..

[60] Omolo M.O., Okinyo D., Ndiege I.O., Lwande W., Hassanali A. (2004). Repellency of essential oils of some Kenyan plants against *Anopheles gambiae*. Phytochemistry.

[61] Makgakga C. (2004). National Herbarium (Pretoria). http://pza.sanbi.org/cannabis-sativa.

[62] Bala A., Rademan S., Kevin K.N., Maharaj V., Matsabisa M.G. (2019). UPLC-MS analysis of *Cannabis sativa* using tetrahydrocannabinol (THC), cannabidiol (CBD), and tetrahydrocannabinolic acid (THCA) as marker compounds: Inhibition of breast cancer cell survival and progression. Nat. Prod. Commun..

[63] Tariq A.L., Reyaz A.L. (2012). Isolation of cannabinoids from the plant *Cannabis sativa* and its potential anticancer activity. Int. J. Drug Dev. Res..

[64] Seltzer E.S., Watters A.K., MacKenzie D., Granat L.M., Zhang D. (2020). Cannabidiol (CBD) as a promising anti-cancer drug. Cancer.

[65] (2021). Invasive Species South Africa (ISSA). http://www.invasives.org.za/legislation/item/594-madagascar-periwinkle-cantharanthus-roseus.

[66] Shikhare S. (2020). *Catharanthus roseus*: A Symbol of Hope for Cancer Patients. Oncol. Cancer. Case Rep..

[67] Deutschländer M.S., Lall N., van de Venter M. (2009). Plant species used in the treatment of diabetes by South African traditional healers: An inventory. Pharm. Biol..

[68] Harshini M., Sheeba L., Selvanayaki M. (2020). Anticancer Activity of *Catharanthus roseus* and *Murraya koenigii*. J. Crit. Rev..

[69] Phama H.N.T., Sakoffc B., Vuonga J.A., Bowyera Q.V., Scarlett J., Christopher M.C. (2018). Screening phytochemical content, antioxidant, antimicrobial and cytotoxic activities of *Catharanthus roseus* (L.) G. Don stem extract and its fractions. Biocatal. Agric. Biotechnol..

[70] Wang L., He H.-P., Di Y., Zhang Y.-T., Hao X.-J. (2012). Catharoseumine, a new monoterpenoid indole alkaloid possessing a peroxy bridge from *Catharanthus roseus*. Tetrahedron Lett..

[71] Wang C.-H., Wang G.-C., Wang Y., Zhang X.-Q., Huang X.-J., Zhang D.-M., Chen M.-F., Ye W. (2012). Cytotoxic dimeric indole alkaloids from *Catharanthus roseus*. Fitoterapia.

[72] Notten A. Kirstenbosch NBG. http://pza.sanbi.org/eucomis-autumnalis.

[73] Bisi-Johnson1 M.A., Obi C.L., Hattori T., Oshima Y., Li S., Kambizi L., Eloff J.N., Vasaikar S.D. (2011). Evaluation of the antibacterial and anticancer activities of some South African medicinal plants. BMC Complement. Altern. Med..

[74] Le Roux L.-N. (2004). Lowveld National Botanical Garden. http://pza.sanbi.org/euphorbia-ingens.

[75] Mofokeng M.M., Hintsa T.A., Amoo S.O., Sehlola D., du Plooy C.P., Bairu M.W., Venter S., Mashela P.W. (2020). Diversity and Conservation through Cultivation of Hypoxis in Africa—A Case Study of *Hypoxis hemerocallidea*. Divers.

[76] Singh Y. (2004). Natal Herbarium, Durban. http://pza.sanbi.org/hypoxis.

[77] Mutshinyalo T.T. (2004). Walter Sisulu National Botanical Garden. http://pza.sanbi.org/pittosporum-viridiflorum.

[78] Madikizela B., McGaw L.J. (2017). *Pittosporum viridiflorum* Sims (Pittosporaceae): A review on a useful medicinal plant native to South Africa and tropical Africa. J. Ethnopharmacol..

[79] Madikizela B., McGaw L.J. (2019). In vitro cytotoxicity, antioxidant and anti-inflammatory activities of *Pittosporum viridiflorum* Sims and *Hypoxis colchicifolia* Baker used traditionally against cancer in Eastern Cape, South Africa. S. Afr. J. Bot..

[80] Poschner S. (2003). Anti-Cancer Effects, Cytotoxicity and Metabolism of Purified Cameroonian Plant Extracts. Master’s Thesis.

[81] Welman M. (2004). National Herbarium, Pretoria. http://pza.sanbi.org/solanum-aculeastrum.

[82] Koduru S.V., Grierson D.S., Van de Venter M., Afolayan A.J. (2006). In vitro antitumour activity of *Solanum aculeastrum* berries on three carcinoma Cells. Int. J. Cancer Res..

[83] Burger T., Mokoka T., Fouché G., Steenkamp P., Steenkamp V., Cordier W. (2018). Solamargine, a bioactive steroidal alkaloid isolated from *Solanum aculeastrum* induces non-selective cytotoxicity and Pglycoprotein inhibition. BMC Complement. Altern. Med..

[84] Koduru S.V., Grierson D.S., Van de Venter M., Afolayan A.J. (2007). Anticancer activity of steroid alkaloids isolated from *Solanum aculeastrum*. Pharm. Biol..

[85] Ngcobo M., Gqaleni N., Chelule P.K., Metse S., Assoungac A. (2012). The Immunomodulatory effects of *Sutherlandia frutescens* extracts in human normal peripheral blood mononuclear cells. Afr. J. Tradit. Complement. Altern. Med..

[86] Gouws C., Smit T., Willers C., Svitina H., Calitz C., Wrzesinski K. (2021). Anticancer potential of *Sutherlandia frutescens* and *Xysmalobium undulatumin* LS180 Colorectal Cancer Mini-tumors. Molecules.

[87] Chinkwo K.A. (2005). *Sutherlandia frutescens* extracts can induce apoptosis in cultured carcinoma cells. J. Ethnopharmacol..

[88] Motadi L.R., Choene M.S., Nonkululeko N., Mthembu M.S. (2020). Anticancer properties of *Tulbaghia violacea* regulate the expression of p53-dependent mechanisms in cancer cell lines. Sci. Rep..

[89] Mabona U., Van Vuuren S.F. (2013). Southern African medicinal plants used to treat skin diseases. S. Afr. J. Bot..

[90] Ayele T.T. (2018). A review on traditionally used medicinal plants/herbs for cancer therapy in Ethiopia: Current status, challenge and future perspectives. Org. Chem. Curr. Res..

[91] Thibane V.S. (2018). Ethnopharmacological Study on Plants Used for Skin Care and Beauty by Some Xhosa Communities. Ph.D. Thesis.

[92] Maroyi A. (2017). Diversity of use and local knowledge of wild and cultivated plants in the Eastern Cape province, South Africa. J. Ethnobiol. Ethnomed..

[93] De Wet H., Fouche G., Van Heerden F.R. (2009). In vitro cytotoxicity of crude alkaloidal extracts of South African Menispermaceae against three cancer cell lines. Afr. J. Biotechnol..

[94] Doughari H.J., Ndakidemi P.A., Human I.S., Benade S. (2011). *Curtisia dentata*: Ethnopharmacological application. J. Med. Plant Res..

[95] Medicinal Plants in the Baviaanskloo. https://www.sederkloof.co.za/page/medicinal-plants.

[96] Molefe N.I. (2013). Anthelmintic, Anticancer and Phytochemical Screening of *Cotyledon Orbiculata*; *Hermannia depressa*; *Nicotiana glauca* and Potassium Permanganate. Master’s Thesis.

[97] Sagbo I.J., Mbeng-Otang W. (2018). Plants used for cosmetics in the Eastern Cape province of South. Pharmacogn. Rev..

[98] Koduru S., Asekun O.T., Grierson D.S., Afolayan A.J. (2006). Isolation of volatile compounds from *Solanum aculeastrum* (Solanaceae). J. Essent. Oil Bear. Plants.

[99] Harris S. Free State National Botanical Garden. http://pza.sanbi.org/tulbaghia-violacea.

[100] Mhalad R. (2014). The Therapeutic Value of Aloe ferox Mill.

[101] de Wet H., Van Heerden F.R., Van Wyk B.-E. (2011). Alkaloidal Variation in *Cissampelos Capensis* (Menispermaceae). Molecules.

[102] Kukula-Koch W., Grabarska A., Łuszczki J., Czernicka L., Nowosadzka E., Gumbarewicz E., Jarząb A., Audo G., Upadhyay S., Głowniak K. (2018). Superior anticancer activity is demonstrated by total extract of *Curcuma longa* L. as opposed to individual curcuminoids separated by centrifugal partition chromatograph. Phytother. Res..

[103] Kuttan R., Bhanumathy P., Nirmala K., George M.C. (1985). Potential anticancer activity of turmeric (*Curcuma longa*). Cancer Lett..

[104] Soyingbe O.S., Mongalo N.I., Makhafola J.J. (2018). In vitro antibacterial and cytotoxic activity of leaf extracts of *Centella asiatica* (L.) Urb, *Warburgia salutaris* (Bertol. F.) Chiov and *Curtisia dentata* (Burm. F.) C.A.Sm—Medicinal plants used in South Africa. BMC Complement. Altern. Med..

[105] Fadipe V., Ishmael N., Opoku A.R. (2015). In vitro evaluation of the comprehensive antimicrobial and antioxidant properties of *Curtisia dentata* (Burm.f) C.A. Sm: Toxicological effect on the human embryonic kidney (HEK293) and human hepatocellular carcinoma (HepG2) cell lines. EXCLI J..

[106] Bouke G., Van der Venter M. (2011). Cytotoxicity and mechanism(S) of action of *Hypoxis* spp. (African Potato) against HeLa, HT-29 and MCF-7 cancer cell lines. J. Med. Plants Res..

[107] Bedane K.G., Brieger L., Strohmann C., Seo E.J., Efferth T., Spiteller M. (2020). Cytotoxic Bufadienolides from the Leaves of *Melianthus major*. J. Nat. Prod..

[108] Srividya A.R., Ganesh S.S. (2010). Antioxidant, antimicrobial and cytotoxic property of *Melianthus major* leaves. J. Glob. Pharma Technol..

[109] Wintola O.A., Afolayan A.J. (2011). Phytochemical constituents and antioxidant activities of the whole leaf extract of *Aloe ferox* Mill. Pharmacogn. Mag..

[110] Mwale M., Masika P.J. (2010). Analgesic and anti-inflammatory activities of *Aloe ferox* Mill. aqueous extract. Afr. J. Pharm. Pharmacol..

[111] Afolayan A.J., Grierson D.S., Kambizi L., Madamombe I., Masika P.J. (2002). In vitro antifungal activity of some South African medicinal plants. S. Afr. J. Bot..

[112] Rahmasaria R., Haruyamaa T., Charyasriwonga S., Nishidaa T., Kobayash N. (2017). Antiviral Activity of *Aspalathus linearis* against Human Influenza Virus. Nat. Prod. Commun..

[113] Mujeeb H. (2019). The Effects of *Aspalathus linearis* (Rooibos Tea) on Nitric Oxide (NO) and Cytokine Activity. Int. J. Heal. Hum. Sci..

[114] Singh D.N., Verma N., Raghuwanshi S., Shukla P.K., Kulshreshthaa D.K. (2008). Antifungal activity of *Agapanthus africanus* extractives. Fitoterapia.

[115] Pellegrini M., Palmieri S., Ricci A., Serio A., Paparella A., Sterzo C.L. (2020). In vitro antioxidant and antimicrobial activity of *Cannabis sativa* L. cv ‘Futura 75’ essential oil. Nat. Prod. Res..

[116] Gajalakshmi S., Vijayalakshmi S., Devi R.S. (2013). Pharmacological activities of *Catharanthus roseus*: A perspective review. Int. J. Pharm. Bio Sci..

[117] Bhutkar M.A., Bhise S.B. (2011). Comparative Studies on antioxidant properties of *Catharanthus rosea* and *Catharanthus alba*. Int. J. Pharmtech. Res..

[118] Barik K., Sao K., Parihar D.K. (2016). Phytochemicals and pharmaceutical panorama of *Catharanthus roseus*. Indo Am. J. Pharm. Sci..

[119] Adedapo A.A., Jimoh F.O., Afolayan A.J., Masika P.J. (2009). Antioxidant properties of the methanol extracts of the leaves and stems of Celtis Africana. Rec. Nat. Prod..

[120] Borquaye L.M., Saah S.A., Adu-Poku D., Adu-Gyamfi L., Bitian K., Bambil W. (2020). Anti-inflammatory, antioxidant and total phenolic content of the ethanolic extracts of *Celtis africana* Burm. f. Curr. Sci. Perspect..

[121] Babajide O.J., Mabusela W.T., Green I.R., Farouk A., Frans W., Iwuoha E.I. (2010). Phytochemical screening and biological activity studies of five South African indigenous medicinal plants. J. Med. Plant Res..

[122] Mukhopadhyay A., Basu N., Ghatak N. (1982). Anti-inflammatory and irritant activities of curcumin analogues. Agents Act..

[123] Park E.J., Jeon C.H., Ko G. (2000). Protective effect of curcumin in rat liver injury induced by carbon tetrachloride. J. Pharm. Pharmacol..

[124] Rasmussen H.B., Christensen S.B., Kvist L.P., Karazami A. (2000). A simple and efficient separation of the curcumins, the antiprotozoal constituents of *Curcuma longa*. Planta Med..

[125] Oyedemi S., Oyedemi B.O., Arowosegbe S.O., Afolayan A.J. (2012). Phytochemicals analysis and medicinal potentials of hydroalcoholic extract from *Curtisia dentata* (Burm.f) C.A. Sm Stem Bark. Int. J. Mol. Sci..

[126] Hulley M.I., Van Vuuren S.F., Sadgrove N.J., Van Wyk B.-E. (2019). Antimicrobial activity of *Elytropappus rhinocerotis* (Asteraceae) against micro-organisms associated with foot odour and skin ailments. J. Ethnopharmacol..

[127] Ramavhoya M.R. (2005). Chemical and Biological Properties of *Euphorbia ingens* E.Mey. Master’s Thesis.

[128] Buwa L.V., Van Staden J. (2006). Antibacterial and antifungal activity of traditional medicinal plants used against venereal diseases in South Africa. J. Ethnopharmacol..

[129] McGaw L.J., J¨ager A.K., Van Staden J. (2000). Antibacterial, anthelmintic and anti-amoebic activity in South African medicinal plant. J. Ethnopharmacol..

[130] Nkomo M., Nkeh-Chungag B.N., Kambizi L., Ndebia E.J., Iputo J.E. (2010). Antinociceptive and anti-inflammatory properties of *Gunnera perpensa* (gunneraceae). Afr. J. Pharm. Pharmacol..

[131] Reid R.A. (2002). Pharmacological Properties of Sterculiaceae. Ph.D. Thesis.

[132] Akinrinde A.S., Afolayan A.J., Bradley G. (2018). Phytochemical composition and antioxidant activities of *Dianthus thunbergii* Hooper and *Hypoxis argentea* Harv Ex Baker: Plants used for the management of diabetes mellitus in Eastern Cape, South Africa. Pharmacogn. Mag..

[133] Akinrinde A.S., Koekermoer T., van der venter M., Bradley G. (2018). In vitro investigation of potential anti-diabetic activity of the corm extract of *Hypoxis argentea* Harv. Ex Baker. Acta Pharm..

[134] Bassey K., Cosa S. (2020). Antiuropathogenic and antioxidant activities of *Hypoxis hemerocallidea* Lam. extracts, and compounds from its taxonomically related species. Arch. Pharm. Pharm. Sci..

[135] Oguntibeju O.O., Meyer S., Aboua Y.G., Goboza O. (2016). *Hypoxis hemerocallidea* significantly reduced hyperglycaemia and hyperglycaemic-Induced oxidative stress in the liver and kidney tissues of streptozotocin-Induced diabetic male wistar rats. Evid. Based Complement. Altern. Med..

[136] Powrie A.H. (1975). Chemical Constituents of *Knoltonia capensis*. Master’s Thesis.

[137] Amoo S.O., Aremu A.O., Moyo M., Van Staden J. (2012). Antioxidant and acetylcholinesterase-inhibitory properties of long-term stored medicinal plants. BMC Complement. Altern. Med..

[138] Ncube B., Finnie J.F., Van Staden J. (2012). In vitro antimicrobial synergism with in plant extract combinations from three South African medicinal bulbs. J. Ethnopharmacol..

[139] Mabona U. (2013). Antimicrobial Activity of Southern African Medicinal Plants with Dermatological Relevance. Master’s Thesis.

[140] Naidoo D., Van Vuuren S., Van Zyl R., de Wet H. (2013). Plants traditionally used individually and in combination to treat sexually transmitted infections in northern Maputaland, South Africa: Antimicrobial activity and cytotoxicity. J. Ethnopharmacol..

[141] Katerere D.R., Eloff J.N. (2005). Antibacterial and antioxidant activity of *Sutherlandia frutescens* (Fabaceae), a reputed anti-HIV/AIDS phytomedicine. Phytother. Res..

[142] Chadwick W.A., Roux S., Van de Venter M., Louw J., Oelofsen W. (2007). Anti-diabetic effects of *Sutherlandia frutescens* in Wistar rats fed a diabetogenic diet. J. Ethnopharmacol..

[143] Maleeha F.-S. (2019). The Anti-Inflammatory Effects of *Sutherlandia frutescens* in a Cell and Animal Model. Ph.D. Thesis.

[144] Moodley K., Joseph K., Naidoo Y., Islam S., Mackraj I. (2015). Antioxidant, antidiabetic and hypolipidemic effects of *Tulbaghia violacea* Harv. (wild garlic) rhizome methanolic extract in a diabetic rat model. BMC Complement. Altern. Med..

[145] Ranglová K., Krejčová P., Kubec R. (2015). The effect of storage and processing on antimicrobial activity of *Tulbaghia violacea*. S. Afr. J. Bot..

[146] PlantZ Africa. http://pza.sanbi.org.

[147] The Plant List. http://www.theplantlist.org.

